# Shoulder sonography: Diagnostic and interventional utility

**DOI:** 10.4103/0973-6042.76958

**Published:** 2010

**Authors:** Joe de Beer, Deepak N. Bhatia

**Affiliations:** Cape Shoulder Institute, Platekloof, Cape Town, South Africa; 1 Department of Orthopaedic Surgery, Seth GS Medical College and King Edward VII Memorial Hospital, Parel, Mumbai - 400 012, India

## INTRODUCTION

Sonography is an effective tool in the evaluation and management of shoulder problems. The utility of shoulder sonography extends across the entire spectrum of shoulder pathology; although most soft tissue structures can be visualized using appropriate techniques, consistent and reliable recognition of pathology requires a reasonable level of skills and experience. magnetic resonance imaging offers an operator-independent and reliable alternative to sonography; however, sonography provides a low-cost office-based imaging method, and both static and dynamic techniques can be performed and interpreted instantly. A concise description of the diagnostic and therapeutic applications of shoulder ultrasonography, as performed by the authors, is presented here.

## DIAGNOSTIC APPLICATIONS OF SHOULDER SONOGRAPHY

### Rotator cuff tears

**Figure 1:**
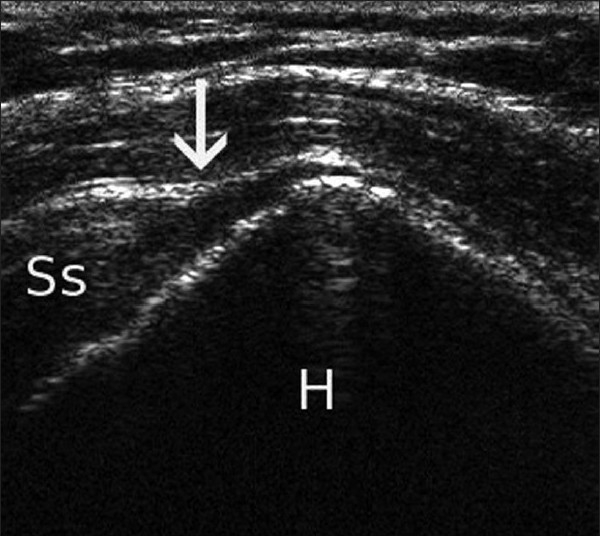
A full-thickness tear (arrow) of the supraspinatus (Ss) is shown (H: humeral head)

**Figure 2:**
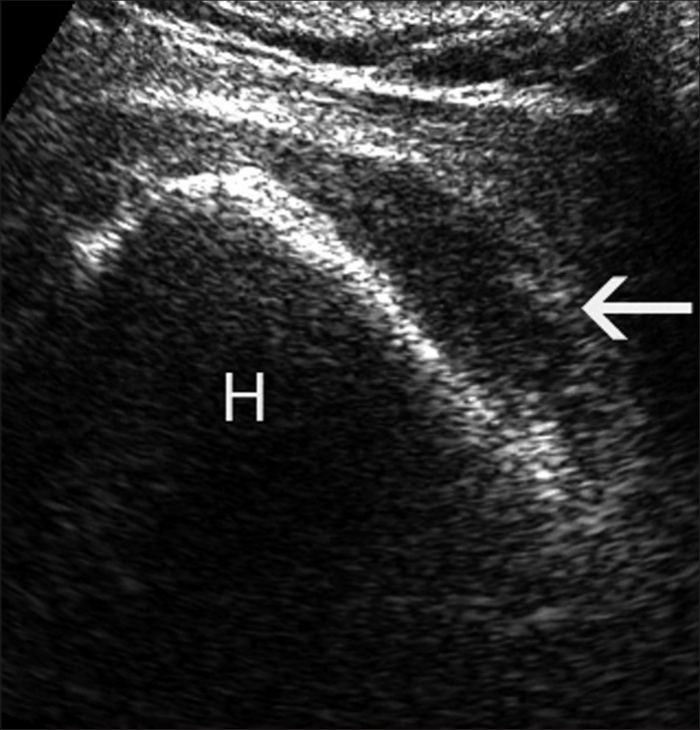
Subdeltoid bursal effusion (arrow) is shown (H: humeral head)

Sonography, in experienced hands, equals the accuracy of magnetic resonance imaging (MRI) for the diagnosis of full thickness rotator cuff tears.[1] Trained shoulder surgeons and radiologists have comparable results in diagnosis of these tears.[2] Correct technique and interpretation permits accurate static visualization of the tendon disinsertion on the greater tuberosity; real-time passive motion of the arm may reveal dyssynchronous motion of the rotator cuff and tuberosity, and a comparison with the contralateral side can be performed [Figure 1]. In addition, bony changes on the surface of the greater tuberosity footprint area may be indirect indicators of rotator cuff disease. The presence of subdeltoid effusions and flattening of the cuff tendon should raise the suspicion of rotator cuff pathology, and this is often a bursal sided partial tear. Articular-sided partial tears may be visualized as hypoechoic or mixed echogenic defects at the articular surface of the cuff. In large tears, the degree of retraction can be assessed. However, fatty infiltration and muscle atrophy have not been as well described on sonography as on MRI. The postoperative integrity of a repaired rotator cuff tear can easily be evaluated by passively moving the arm, and synchronous movement of the cuff with the humeral head indicates an intact repair. The presence of a subacromial/subdeltoid effusion in the follow-up evaluation may indicate inflammation or may even be an early warning of postoperative infection [Figure 2]. If the amount of effusion is of concern, ultrasonography-guided needle aspiration of the fluid can be done for laboratory analysis. Lytic areas around the bone anchors may also be an early sign of postoperative infection. Other aspects include visualization of dislodged bioabsorbable anchors which are not visible on radiographs.

### Calcific tendinitis of the rotator cuff

**Figure 3:**
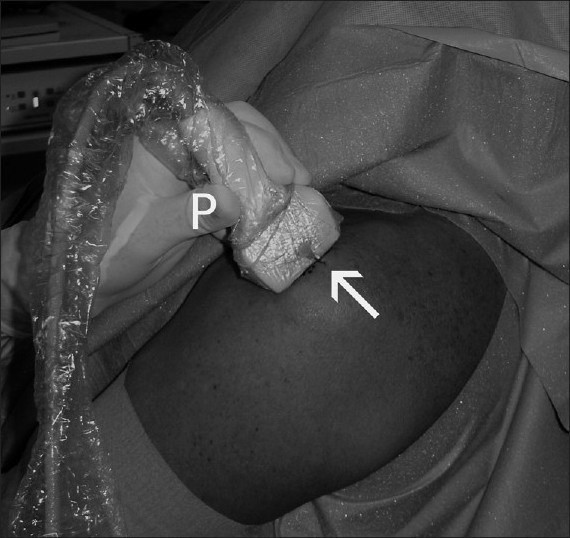
Intraoperative use of ultrasound is shown for needle localization (arrow) of a calcific deposit. A sterile cover is used with a standard superficial probe (P) and sterile ultrasound gel

Calcific deposits of the rotator cuff are well seen on ultrasonography, and their exact location and size can be evaluated in the all dimensions. Ultrasonography is a well-established treatment modality for this condition both with injections and “needling”. Shoulder ultrasound is also useful for intraoperative localization of the calcific deposit during arthroscopic calcific deposit excision [Figure 3].

### Acromioclavicular joint degeneration

**Figure 4:**
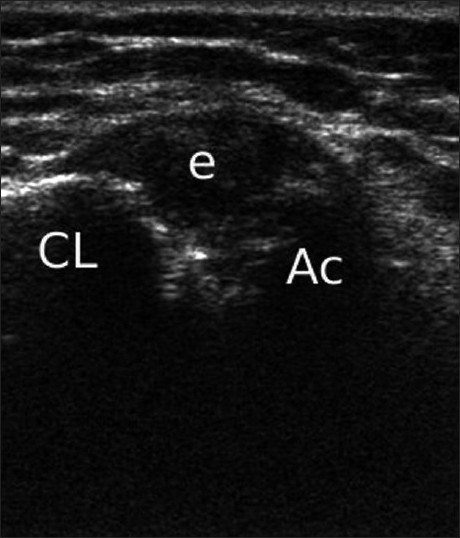
Acromioclavicular joint effusion (e) is shown (Ac: acromio, CL: clavicle)

Ultrasonography is useful in the diagnosis of the acromioclavicular joint (ACJ) involvement as a cause of pain in the shoulder. The presence of an effusion of the ACJ is a valuable indicator of the ACJ being symptomatic [Figure 4]. The size of the effusion can easily be compared to the contralateral side and the surface area of the effusion be measured. Ameliorative injection testing of the ACJ with local anesthetic agents under sonographic control is of great value in the diagnosis of ACJ pathology.

### ACJ subluxations

The degree of ACJ subluxation can be determined accurately on ultrasonography due to its three-dimensional advantage. The mobility of the distal end of the clavicle can also be determined by exerting pressure on it. Posterior subluxation of the ACJ, often difficult to visualize on plain radiographs, can also be diagnosed.

### Disorders of the long head of the biceps tendon

Effusions in the bicipital groove can be demonstrated with accuracy. The probe should be moved distally down the arm due to the fact that these effusions are often more pronounced distally due to gravitation. Subluxation of the biceps out of the groove is well seen, and if necessary, passive rotational movements can demonstrate the subluxation in real time. Dynamic assessment of an hour-glass enlargement of the long biceps tendon has also been recently described.[3] Injections into the groove can be given accurately for ameliorative testing of bicipital pain. Bony morphology of the bicipital groove is well demonstrated on ultrasonography, and disorders of the bony anatomy of the bicipital groove (irregularities, narrow dimensions, etc.) can be well seen.

### Osteoarthritis of the glenohumeral joint

The integrity of the rotator cuff, which has enormous bearing on the treatment options (anatomic prosthesis or reverse prosthesis), can be evaluated. Congruency of the joint can be seen; the typical posterior subluxation of the humeral head is apparent when placing the probe on the posterior aspect of the joint.

### Shoulder instability and labral tears

The presence and size of a Hill-Sachs lesion can be seen by placing the probe on the posterior aspect of the humeral head. Locked posterior dislocation is well visualized. Dynamic testing is useful in the diagnosis of glenohumeral subluxation and labral lesions.

### Fractures

Minimally and undisplaced fractures of the tuberosities of the humeral head that are not visible on radiographs can often be diagnosed on sonography. Fractures of spine of the scapula are difficult to visualize on radiographs and can easily be seen on sonography. Early callus formation can be detected before seen on radiographs.

### Thoracic outlet syndrome

Sonography is a useful tool to evaluate potential reasons for nerve compression; in some cases, hypertrophy of the scalene muscles with resultant compression of the brachial plexus may be detected.[4]

## DIAGNOSTIC AND THERAPEUTIC INTERVENTIONS UNDER SONOGRAPHIC CONTROL

### Ultrasound-guided injections

The shoulder joint is compartmentalized into three levels (glenohumeral, subacromial, and acromioclavicular joints) and ultrasound-guided injections can be given accurately into each of these for therapeutic or diagnostic purposes. In addition, bicipital groove injections and injections into the suprascapular/spinoglenoid notches may be given using sonographic control.

### “Needling” procedure for calcific tendinitis

Correct technique is necessary to achieve a satisfactory success rate to eliminate calcific deposits from the rotator cuff without having to resort to surgical procedures. The senior author’s technique involves the use of two large gauge needles and “flushing” out the calcium from the deposit site with sterile saline.

### Aspiration of peri-labral cysts

**Figure 5:**
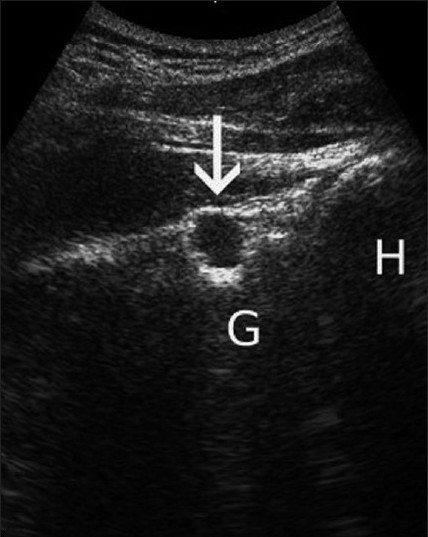
The spinoglenoid notch is shown and a cyst (arrow) is visualized in this region (G: Glenoid; H: Humeral head)

These cysts often result from posterior labral tears and cause impingement of the suprascapular nerve in the spinoglenoid notch. Sonography-guided aspiration of such cysts is a simple procedure with a reasonable success rate and may be followed by surgical evacuation and labral repair if the aspiration fails to resolve the symptoms [Figure 5].

### Injection into the defect of os acromiale

The presence of an os acromiale can usually be seen on radiographs. The dilemma is often to decide if pain in the shoulder is caused by the “non-union” site of the acromion, secondary effect of non-outlet impingement or other causes. A useful diagnostic test is to inject local anesthetic solution into the defect with sonography control and noting the pain relief.

### Thoracic outlet syndrome

Interventional procedures for the thoracic outlet compression syndrome have been suggested. These involve ultrasound-guided injections of local anesthetic agents into the scalene muscles, and the resultant pain relief from temporary paresis of these muscles is diagnostic. Botulinum toxin injection into the scalene muscles, again with sonographic guidance, may be of great benefit for temporary pain relief over a few months in this condition. Such a positive response serves as a predictor of a good surgical outcome for scalenotomy procedures.

### Pectoralis minor injections

**Figure 6:**
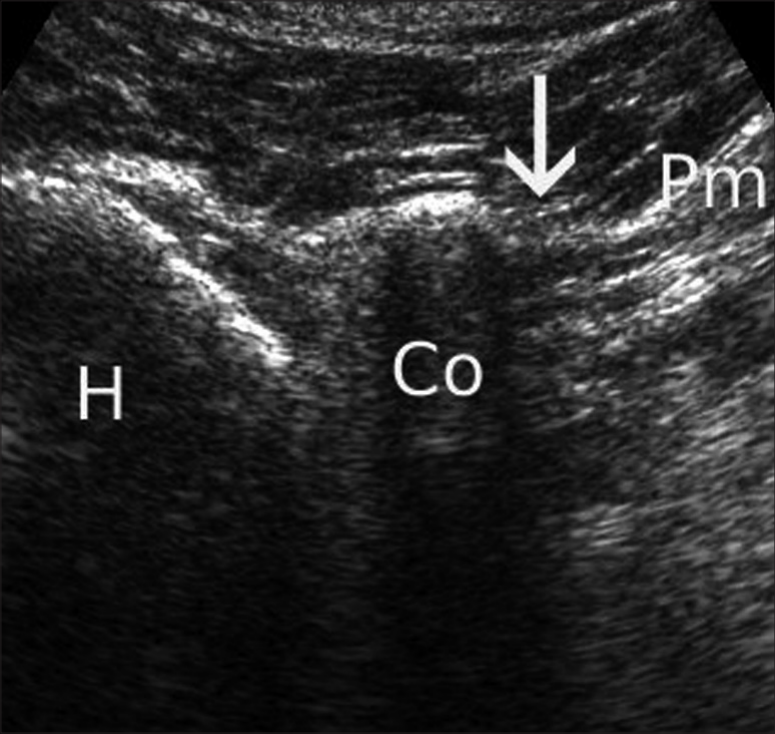
Sonographic visualization of the pectoralis minor insertion (arrow) is shown (Pm: Pectoralis minor muscle; Co: Coracoid process; H: humeral head)

Insertional tendinopathy of the pectoralis minor muscle (bench-presser’s shoulder) can be effectively treated with a single ultrasound-guided injection of a solution of a local anesthetic agent and a steroid.[5] The technique for this procedure involves sonographic localization of the pectoralis minor tendon, and care should be taken to avoid inadvertent injection into the underlying neurovascular structures[Figure 6]

### Brachial plexus blocks

Ultrasonography-guided nerve blocks are well described in the perioperative management of shoulder pain.[6,7]

From the above, it is evident that ultrasonography is an indispensable tool for advanced shoulder diagnosis and treatment. Several centers involved in the management of shoulder problems now use office-based sonography in conjunction with clinical examination in a “one-stop clinic” approach. Future developments in sonographic resolution, doppler sensitivity, and multidimensional imaging will provide new avenues for research and management of various shoulder problems.
